# Master statistical analysis plan: attractive targeted sugar bait phase III trials in Kenya, Mali, and Zambia

**DOI:** 10.1186/s13063-023-07762-7

**Published:** 2023-11-29

**Authors:** Joshua Yukich, Thomas P. Eisele, Feiko terKuile, Ruth Ashton, Sarah Staedke, Angela F. Harris, Paul C. D. Johnson, Sophie Sarrassat, Maia Lesosky, John Bradley, Immo Kleinschmidt, Megan Littrell, Attractive Targeted Sugar Bait Phase III Trial Group

**Affiliations:** Tulane University School of Public Health and Tropical Medicine, New Orleans, USA

**Keywords:** Malaria, Vector control, Cluster randomized control trial, Statistical analysis plan

## Abstract

This manuscript is a master statistical analysis plan for each of three-cluster randomized controlled trials to evaluate the efficacy of attractive targeted sugar baits (ATSB) described in an already published protocol. The master SAP contains an overarching plan for all three trials, which can be adapted to trial-specific circumstances. The primary objective of the trials is to evaluate the efficacy of ATSB in the presence of universal vector control coverage with insecticide-treated nets (ITN) or indoor residual spraying (IRS) after two transmission seasons on clinical malaria incidence as compared with universal vector control coverage with ITN or IRS alone. The primary outcome measure is the incidence rate of clinical malaria, assessed in cohorts aged 12 months to less than 15 years (≥ 5 years to 15 years in Mali) during monthly follow-up visits. The primary unadjusted analysis will be conducted on the intention-to-treat analysis population without adjustment for any anticipated confounding variables. The primary outcome will be analyzed using a multi-level model constructed on a generalized linear model framework with a Poisson likelihood and a log link function. Random intercepts will be included for each study cluster and a fixed effect for study-arm. The analyst will be blinded to study arm assignment. Several secondary outcomes will be analyzed, as well as a pooled analysis (individual patient data meta-analysis) across the three trial sites. Additionally, a standard meta-analysis is expected to be conducted using combined data from all sites.

**Supplementary Information:**

The online version contains supplementary material available at 10.1186/s13063-023-07762-7.

## Introduction

### Background and rationale

Highly effective interventions against malaria vectors that preferentially feed on humans late at night and rest inside houses have been developed and implemented at scale. Their effectiveness is a function of the fact that they specifically target indoor-biting and indoor-resting mosquitoes, which are often the same mosquito species comprising the bulk of the vectorial system.

However, several mosquito species have evolved high levels of resistance to the insecticides used in long-lasting insecticide-treated nets (LLINs) and indoor residual spraying (IRS) as a result of prolonged exposure through the scale-up of these interventions. There is increasing concern that this insecticide resistance is undermining the effectiveness of these interventions. Furthermore, malaria vectors exhibit different behavioral characteristics, such as outdoor and daytime biting, that compromise the effectiveness of existing vector control strategies.

In addition to the biological need for female *Anopheles* species to take a blood meal to obtain the protein necessary for egg production, all *Anopheles* must feed regularly and frequently on liquid and carbohydrates (sugars) to survive. Mosquitoes are guided to sugar sources by chemical attractants. The ATSB (Attractive Targeted Sugar Bait) is designed specifically to attract the mosquito with a source of liquid and sugar and includes an ingestion toxicant to then kill the mosquito. Using sugar sources to attract mosquitoes to an ingestion toxicant is a relatively simple and inexpensive strategy that has been shown to be highly efficacious for mosquito control in a limited number of trials.

**Fig. 1:**
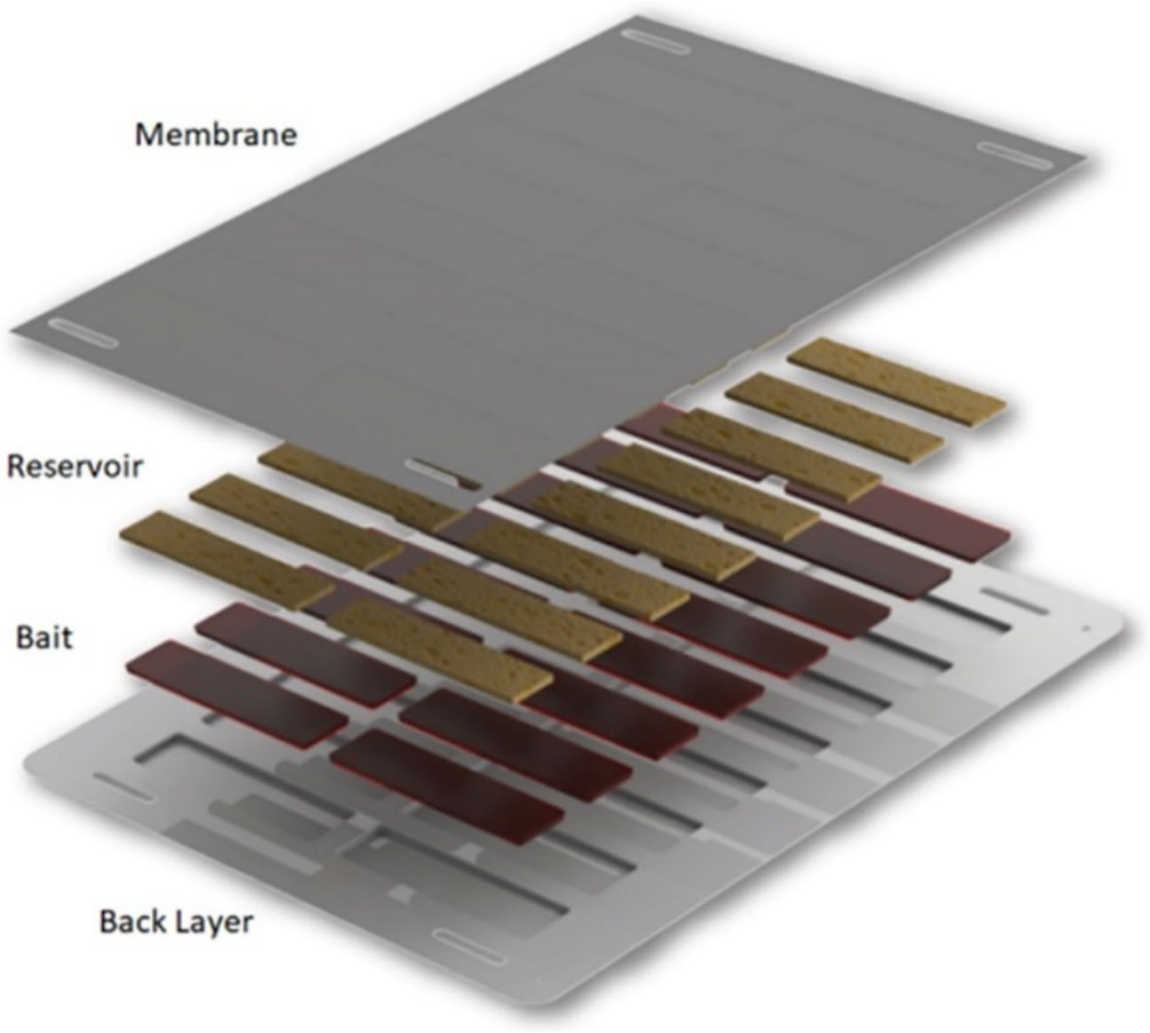
ATSB design

Westham Co. developed a bait station that contains a plant-based mosquito attractant, sugar as a feeding stimulant, and an active ingredient (the neonicotinoid, dinotefuran) to kill the foraging vectors. The bait additionally contains a commonly used bittering agent called Bitrex (https://www.bitrex.com/en-us) that deters mammalian consumption of the bait. The bait station has a protective membrane that covers and protects the bait from rain and dust, but that allows mosquitoes to feed through it (see Fig. 1). Durability studies conducted in Mali, Kenya, and Zambia in 2019–2021 showed that the Westham ATSB can remain effective in the field for at least 6 months. The protective membrane allows mosquitoes to feed, but it serves as a barrier to pollinators. Field studies to-date have shown that the ATSB has a minimal impact on non-target organisms. This includes evidence specifically for the toxicant that will be used, dinotefuran. An initial environmental assessment and subsequent field trials in Mali have demonstrated that when deployed within the ATSB, the toxicant does not pose safety risks to non-target organisms, including pollinators and humans (unpublished data, personal communication with GC Muller).

The Westham ATSB was selected based on results from early testing of bait stations in Israel and Mali. In these studies, bait stations with a food dye marker (without toxin) established that large proportions (> 25%) of the mosquito population were marked daily by the food dye [1]. Proof of concept studies for the impact on mosquito vectors in Mali began in 2015 with a collaborative team from Hebrew University, University of Bamako, University of Miami, Tel Aviv University, and University of Haifa. Research beginning in early 2017 incorporated the toxicant dinotefuran into the bait stations. Early entomological results indicate that outdoor use of ATSBs reduces vector abundance and skews the adult age distribution towards younger mosquitoes which are not infective [1, 2]. Field studies in Mali concluded in early 2018 demonstrated the impact of the ATSB on entomological measures and established an optimal deployment pattern for the local setting [1, 2]. This deployment protocol of two ATSBs installed on opposite exterior walls of sleeping structures at a height of 1.8 m was associated with a target mosquito daily feeding rate of at least 30%. The drastic reduction in mosquito density, particularly of older females, proportion of sporozoite-infected females, and entomological inoculation rate suggest that the ATSB can significantly reduce malaria parasite transmission [2].

Modeling of entomological ATSB study data suggests that ATSBs could markedly reduce mosquito populations across a range of different transmission intensities and should have great potential when used in combination with other indoor vector control tools.

The World Health Organization Vector Control Advisory Group (VCAG) reviewed these data and recommended the evaluation of the potential of the Westham ATSB to reduce clinical malaria incidence in different transmission settings in sub-Saharan Africa. This SAP is intended to serve as a master SAP for each of three trial sites in Kenya, Mali, and Zambia. Three harmonized clinical trials will use this master plan as the basis for site-specific SAPs which may contain minor modifications to adhere to site-specific nuances, including but not limited to, changes in covariables included in analysis or definitions and cutoffs of said variables and summary measures. While the intent of the harmonization is to largely ensure that the trial analysis is conducted comparably and identically, the site-specific SAPs will require minor modifications [3].

## Research questions and hypotheses

### Primary research question

1. Is outdoor deployment of ATSBs plus universal vector control coverage (LLIN or IRS) more effective than universal vector control coverage alone at reducing cohort-based clinical malaria incidence over a 2-year period?

### Secondary research questions

(2) Is deployment of ATSBs associated with a reduction in community parasite infection prevalence?
(3) Is deployment of ATSBs associated with a reduction in passively detected confirmed malaria case incidence?
(4) Is deployment of ATSBs associated with a decline in malaria vector abundance (particularly among older females), longevity of vector mosquitoes (parity status), sporozoite rates, and EIR?
(5) What are the barriers to high ATSB coverage?
(6) Does ATSB deployment affect LLIN use?
(7) What is the cost and cost-effectiveness of outdoor ATSB deployment as a vector control intervention?

## Description of research objectives

### Primary objective

1. To evaluate the efficacy of ATSB deployment in the context of universal vector control coverage (IRS or LLIN) coverage after two transmission seasons in population-based cohort clinical malaria incidence as compared with universal coverage with standard vector control alone.

### Secondary objectives

(2) To evaluate the efficacy of ATSB deployment in the context of universal vector control coverage (IRS or LLIN) on community parasite infection prevalence as compared with universal coverage of vector control alone.
(3) To evaluate the efficacy of ATSB deployment in the context of universal vector control coverage (IRS or LLIN) on passively detected confirmed malaria case incidence as compared with universal coverage of vector control coverage alone.
(4) To assess a minimum set of entomological outcomes (parity, mosquito abundance, human landing rate, entomological inoculation rate) that measure ATSB efficacy in reducing the target vector population and transmission.
(5) To assess the acceptability of ATSBs by communities and other stakeholders. This includes the identification of potential barriers to uptake and consistent ATSB coverage, together with an assessment of ATSB impact on coverage and use of existing malaria control interventions (e.g., LLIN use, treatment-seeking behavior).
(6) To estimate the cost and cost-effectiveness of deploying ATSBs for malaria control.
(7) To assess the safety of ATSBs on humans by monitoring adverse effects in communities where ATSBs are deployed compared to the control.

## Study methods

### Trial design

An open-label two-arm cluster randomized controlled trial (CRCT) design will be used comparing ATSB + universal coverage with a WHO core VC intervention vs universal coverage with VC alone (in the context of other standard-of-care malaria interventions appropriate to the local context including case management, administration of vaccines, seasonal malaria chemoprophylaxis, where applicable). The trial will follow a group-sequential design [4] with one (two in Kenya) potential interim analysis. Three stand-alone superiority CRCTs will be conducted, one in each of Kenya, Mali, and Zambia with design and methods standardized across sites. Each trial is expected to have sufficient power (≥ 80%) to answer the primary research questions in that setting. Universal VC (mainly using LLIN) will be ensured in both arms prior to the start of the study. The intervention arm A will receive ATSBs for up to 2 years. The control arm B will receive universal vector control coverage. Both study arms will receive any other standard-of-care malaria control and prevention interventions such as treatment of uncomplicated malaria with artemisinin combination therapies which may vary from location to location (e.g., including seasonal malaria chemoprophylaxis and RTS,S vaccination in some sites).

### Randomization

Restricted randomization was used to allocate study clusters to intervention and control arms with a balance between study arms on key baseline characteristics, including the primary outcome. Steps one through seven below were carried out by an independent statistician in collaboration with a member of the study team who was *not* responsible for trial implementation. Randomization was conducted independently for each trial. The steps for randomization are as follows:

1. Establish balance criteria. The factors described in Table 1 below may be considered for suitability as restriction criteria. This list is suggestive rather than prescriptive and specific criteria and restriction limits will vary by study site. Criteria for determining balance will be varied during the restricted randomization process to both ensure balance and the validity and lack of bias in study design. In the Zambia site for example, randomization lists were designed to include balance on malaria prevalence by RDT, whether or not entomological data collection was to be conducted in the cluster, bed net use reported in a baseline cross-sectional survey, and the use of indoor residual spraying in the cluster.
2. Generate a list of at least 100,000 randomizations (Allocation sequences)
3. Check randomizations (allocation sequences) against balance criteria and drop those that do not meet balance criteria
4. Assess the number of randomizations (allocation sequences) remaining. If fewer than 10,000 acceptable randomizations (sequences) remain, stop and relax restriction criteria. If a high proportion of allocation sequences remain (e.g., > 90%), consider tightening balance criteria.
5. Test the remaining set of potential randomizations (allocation sequences) for validity, specifically that all clusters are being independently assigned to study arms (i.e., check that no two clusters are disproportionately jointly assigned to the same or disproportionately to opposite arms).
6. Randomly choose a randomization (allocation sequence).
7. Flip a coin to determine if arm A or arm B is ATSB or control.

**Table 1:** Covariates to be considered for restricted randomization

Covariate/endpoint	Restriction criteria	Data source	Analytic method
Malaria disease incidence	Difference in mean baseline clinical case incidence between trial arms (size of difference to be assessed when data are available)	Baseline cohort	Difference in baseline disease incidence of cluster summaries between study arms
Bednet use	Difference in mean proportion of persons slept under any net night before survey between trial arms ≤ 5 percentage points	Baseline survey	Difference in means of cluster summaries of proportion of persons of all ages slept under any net night before survey between arms
Population	Total population size of larger trial arm no more than 10% larger than smaller arm	Enumeration datasets	Sum (pop size of clusters arm large)/sum (pop size of clusters arm small) less than 1.10
Urbanization^a^	Number of urban clusters in each arm nearly balance	Census data using national classification (alternatively remotely sensed classification could be used (GRUMP/WorldPop))	*N* in arm A ± 1 of *N* in Arm B
Housing density^a^	Difference in mean housing density between trial arms ≤ 0.3 SD of overall cluster level housing density	Enumeration + cluster boundaries GIS files Or Remote sensed data (GRUMP/WorldPop) plus Cluster boundaries GIS	SD (cluster estimates of housing densities) × 0.3 ≥|mean (cluster estimates housing density Arm a) – mean (cluster estimates of housing density Arm b)|
HF location	Number of clusters with a primary care facility nearly balanced across arms	Study team documentation	*N* in arm A ± 1 of *N* in Arm B
Altitude	Differences in mean altitude of cluster centroids between trial arms ≤ 0.3 SD of overall cluster level mean altitude	Digital Elevation Model (ASTER) combined with (GIS) shape files for cluster boundaries	SD (cluster estimates of altitude) × 0.3 ≥|mean (cluster estimates of altitude Arm a) – mean (cluster estimates of altitude Arm b)|
Entomological data collection	Number of clusters with entomological data collection planned is exactly equal across study arms	Study team self-report	*N* in arm A = *N* in arm B

^a^Either urbanization or housing density will be selected; these variables are likely collinear

After allocation, the intervention will be implemented in the entire ATSB arm according to the assignment. Allocation of study arms will not be blinded to the participants, the deliverers of the intervention, or the main investigators (but will be to lab workers carrying out tests on blood samples and mosquitos). Sham bait stations will not be used in control areas.

### Sample size

Full details of the sample size calculations are contained in the trial master protocol and study site-specific protocols; the sample size determination is presented here in summary form (Tables 2, 3, and 4).

**Table 2:** Sample size for incidence

	Mali	Kenya	Zambia
Clusters per arm (overall)	38 (76)	35 (70)	35 (70)
Trial duration in calendar years (seasonality of FU) (total FU per participant time in months)	2 years (8-month seasons) (16 months FU)	2 years (12-month seasons) (24 months FU)	2 years (6-month seasons) (12 months FU)
α (type 1 error probability for 2-year trial)	0.05 (Haybittle-Peto with one interim analysis)	0.05 (Haybittle-Peto with two interim analyses at approx. 50% and 75%)	0.05 (Haybittle-Peto with one interim analysis)
Power	88%	80%	80%
Baseline incidence of clinical malaria in the target age group	0.40 events per person-year (based on 0.6 incident events during an 8-month malaria season) (5y– < 15y)	0.845 events per person-year during a 12-m malaria transmission season (1y– < 15y)	0.50 events per 6-month malaria season (Jan–Jun) (1y– < 15y)
Reduction in baseline incidence (incidence rate ratio = 0.70)	30%	30%	30%
Coefficient of variation	0.40	0.40	0.40
Assumed loss of person time, including true LTFU plus loss due to exclusion of person time following each treatment with AL	20%	20%	34%
Total person-years required (number enrolled per cluster before loss-to-follow-up)	3850 person-years (obtained by enrolling 38 individuals per cluster to account for loss-to-follow-up, followed for a total of 16 months)	1260 person-years (obtained by enrolling 13.5 individuals per cluster to account for loss-to-follow-up, followed for a total of 24 months)	1610 person-years (obtained by enrolling 35 individuals per cluster to account for loss-to-follow-up, followed for a total of 12 months)

**Table 3:** Sample size for prevalence outcomes

	**Mali**	**Kenya**	**Zambia**
Cluster per arm	38	35	35
α (2-tailed)	0.05	0.05	0.05
Power	90%	80%	90%
Baseline parasite prevalence measured by RDT among participants aged 6 months and older	50%	29.0%^a^	50.0%
Reduction in baseline prevalence	30%	30%	30%
ICC = intracluster correlation coefficient (coefficient of variation)	0.16 (cv = 0.4)	0.05	0.10
Non-response	20%	20%	20%
Sample size per cluster (accounting for non-response)	25 (32)	24 (30)	16 (20)
Total sample size per survey round/year (accounting for non-response)	1900 (2432)	1680 (2100)^b^	1120 (1400)

^a^Prevalence estimates are for individuals aged ≥ 1 month (Kenya only)

^b^Per year in Kenya as trial uses a continuous survey approach

**Table 4:** Sample size and power characteristics of each trial for entomological measures

	**Kenya**	**Zambia**	**Mali**
Clusters per arm	8 (16 total)	10 (20 total)	15 (30 total)
α	0.05 (two-tailed)	0.05 (two-tailed)	0.05 (two-tailed)
β	0.20 (> 80% power)	0.20 (> 80% power)	0.20 (> 80% power)
Number of months of sampling per cluster	12	8	24
Number of sampling sites (households) per cluster per month	10	10	10

## Case incidence cohort

The sample size calculations for the case incidence cohort were calculated using the formula for cluster randomized trial event rates with a person-time denominator [5]. Assumptions utilized in the calculations are summarized below. These assumptions are based on data from similar studies conducted in comparable settings for each study site. In each case, the calculation was completed for the person-time required to demonstrate superiority with a 30% reduction in cumulative clinical case incidence of malaria over a 2-year period. Note that cohort follow-up time differs across the sites. A seasonal cohort will be implemented in Mali (8 months of follow-up per study year) and Zambia (6 months of follow-up per study year). In Kenya, the cohort study will run continuously for the 2-year period,however, the cohort will be rotated every 6 months (i.e., each individual will be followed for up to 6 months) in the first year but will not be rotated (e.g., each individual will be followed for 1 year in the second year).

## Cross-sectional household survey

The sample size calculations for the parasite prevalence surveys were calculated using the formula for cluster randomized trial proportions [5] using PASS 15 Sample Size Software (©NCSS, Kaysville, Utah) for Kenya and Zambia, and R (© The R Foundation) for Mali.

### Passive case detection

Data from all health facilities regarding people of all ages will be used to calculate confirmed malaria case incidence in the intervention and control clusters in Mali and Kenya. In Kenya, specific dates for data collection will be specified in the study site-specific SAP and protocol in terms of timing of data collection/analysis start after ATSB deployment (i.e., a wash-in period of ~ 2 weeks after ATSB deployment may be included and should be precisely pre-specified in Kenya site specific SAP). In Mali, facility-based assistants use electronic tablets with a custom application for collecting case data. Data will be transmitted weekly to the field data manager. In Kenya, health care data are entered into ScanForm (https://about.scanform.qed.ai/) registers at facilities and by community health workers. Individually identifiable data will not be extracted or collected by the trial staff for this outcome.

### Entomological endpoints

Entomological monitoring will be conducted monthly in a sub-set of clusters throughout the course of the trials. While a number of secondary endpoints will be based on these collections, power and sample size calculations in terms of the number of entomology clusters, number of participating households, and number of nights of collections were based specifically on parity status of female mosquitoes, which is a proxy for the daily female vector mosquito survival. Sample size calculations/power analyses were carried out separately for each trial site and are presented as follows. The power to detect the effect of the ATSB intervention on the non-parous rate (NPR) was estimated by analysis of 1000 simulated trial data sets. The effect of the intervention was assumed to be a reduction of daily survival probability from 80% in the control arm to 75% in the intervention arm, equivalent to an increase in NPR from 48.8 to 57.8% based on the formula from Davidson (Davidson, 1954). Each data set was simulated from a generalized linear mixed-effects model (GLMM) with a binomial response, which was the number of non-parous females counted out of the total number of females collected. Variation in NPR between clusters, households, and months was simulated as normally distributed random effects on the logit scale. The household random effect variance of 0.18 was estimated from pilot data collected in Mali. The cluster random effect was estimated from the same data, but the upper 50% confidence limit of 0.04 was used in preference to anti-conservatively using the point estimate of zero. The inter-month variance was set at 1, giving a monthly mean NPR range of approximately 0.2 to 0.8. The total number of female mosquitoes trapped per household per night was sampled from a simulated Poisson distribution with a mean catch of 2.5 females. Inter-cluster and inter-month variation in the number trapped was simulated as normal random effects on the log scale with variances of 0.16 (equivalent to a coefficient of variation of 0.4) and 0.5 (giving a monthly mean catch range of approximately 0.5 to 6) respectively. The number of clusters to be included per arm of the study to achieve at least 80% power with a 0.05 two-tailed alpha is shown in the table below (along with several other assumptions).

### Framework

The trials are planned under a superiority framework. The comparisons will consist of two-sided tests of the null hypothesis of no difference in efficacy between the ATSB (intervention) arm and the control arm. All primary comparisons will consist of comparisons of the outcome in the intervention arm vs. the outcome in the control arm.

### Statistical interim analyses and guidance

One interim analysis is planned in Mali and Zambia. In Kenya, an additional (second) interim analysis is planned because this trial has a 6-month-longer follow-up than the other two trials because transmission occurs throughout the year in Kenya. In Kenya, the interim analyses will be event, rather than time, driven. In Mali and Zambia, the interim analysis will be conducted at the end of the first transmission season in the first year.

In Kenya, interim analyses will occur either after 50% and 75% of person-time have been completed (i.e., after about 1 and 1.5 years respectively), or after 50% (*n* = 415) and 75% (*n* = 622) of the total number of expected primary outcome events over 2 years in the control arm (*n* = 829) have occurred (whichever comes first). The number of events will be tracked by an independent statistician. In Zambia and Mali, an interim analysis will be conducted after the first transmission season regardless of the total number of events.

The interim analysis will consider a stringent rule in each site based on the Haybittle-Peto boundaries to preserve the overall two-sided type I error rate for efficacy at the *α* = 0.05 level at the final analysis. As such, each interim analysis will use an *α* = 0.001 thereby reducing the probability of type-I error to less than 1 per 1000. The final null-hypothesis significance testing will be conducted with standard alpha levels of 0.05 because of the stringent type-I error criteria proposed for the interim analyses. Because of the stringent* α* levels applied and the smaller sample sizes expected at interim analysis, the power to detect the effect is expected to be low, meaning that a positive interim result is only expected in the case in which the effect size is much larger than expected, variance in outcome data is low, the incidence of disease in the reference arm is much higher than anticipated, or a combination of these factors occurs.

Each study site DSMB will be responsible for determining when an interim analysis is required per trial rules which is automatic in Mali and Zambia at the end of year one. If an interim analysis is indicated, an independent statistician will, in collaboration with the DSMB, conduct formal tests of the study data against the following rules:

Firstly, the trial statistician will provide the independent statistician and DSMB with a dataset prepared for analysis with a dummy treatment code. The independent statistician and DSMB statistician would replace the dummy random treatment code with the actual allocation code and conduct the analysis. Finally, after reviewing the analysis output and verifying the results, the independent statistician in collaboration with the DSMB would summarize the findings in a report addressed to the other members of the DSMB.

### Overwhelming benefit rule

The DSMBs of each trial may consider recommending an early submission of the ATSB dossier for overwhelming benefit if a test of the null hypothesis that the cumulative clinical incidence of malaria in the intervention arm in the intention to treat analysis population is lower than the cumulative clinical incidence in the intention to treat analysis population of the control arm. The null hypothesis was equal incidence between the two arms; the interim analysis assumes a two-sided test of the null hypothesis at a significance level of *α* < 0.001. This test will be conducted using a variance component regression model with a Poisson likelihood and a log link function which includes random cluster-level intercepts. The regression will include a fixed effect for study arm, and the hypothesis will be tested by testing that the incidence rate ratio associated with this covariate is not significantly different than 1 with a *p*-value < 0.001. The DSMB has been tasked with only making a recommendation about early referral of the trials to the vector control advisory group (VCAG) at the World Health Organization. This recommendation is only expected after the results of two trials show significant benefit, either in the interim or in the final analysis. As such the independent statistician working in collaboration with each trial site will advise the DSMB of each trial site on the results of the interim analysis as well as results of interim or final analyses of the other trials. The DSMB can thus include the interim results from other sites in their consideration of recommending an early referral to the VCAG.

A DSMB recommendation to discontinue the trial will not be based on the results of this statistical test. The DSMB can advise continuing the trial even if statistically the boundary is crossed, e.g., in order to continue collecting more epidemiological, entomological, or safety information or data for further sub-group analyses. It is the intent of the investigators to continue the trial even in the case of an early efficacy demonstration across more than one site since there is an expectation of significant heterogeneity in the effect of ATSB across entomological settings.

### Stopping for harm

The trials do not include formal stopping rules based on harm, because the intervention is not targeted to humans and the expected risk to trial participants is expected to be minimal; thus, formal harm-based stopping rules are not needed. However, this does not preclude the DSMB from stopping the trial for harm should unforeseen consequences of the ATSB or trial procedures lead to harm. For example, deliberate abuse or misuse of the ATSB products or unforeseen non-target insect impacts could lead to harm which causes trial stoppage.

### Timing of final analysis

Should no early stopping rule be invoked and the trials continued after each interim analysis, then the final analysis per trial (country) will be conducted collectively at the end of two seasons/years. This analysis will occur at the site (trial) level. A final pooled individual participant data (IPD) analysis and meta-analysis of trial outcomes will be conducted collectively after the termination of the trials in all sites.

## Timing of outcome assessments

### Primary and secondary efficacy outcomes

#### Primary outcome

The primary outcome measure is the incidence rate of clinical malaria defined as history of fever or a measured temperature ≥ 37.5 °C and a positive malaria rapid diagnostic test (RDT) (the definition is specified in full in a later section). This will be assessed among people aged 12 months to less than 15 years (≥ 5 to 15 years in Mali). These outcomes will be ascertained through follow-up visits. Visits will be conducted within ± 5 days for true monthly intervals and specific follow-up time between visits will be computed to the nearest one day.

#### Secondary outcomes

1. Prevalence of malaria infection among participants aged 6 months and older, detected by RDT. This outcome will be assessed annually cross-sectionally (or through a rolling prevalence survey in Kenya). For the cross-sectional analyses (Zambia, Mali), measurement will occur in each member of the study sample within an approximate 1-month (30-day) observation window.
2. Incidence rate of passively reported clinical malaria among participants of all ages, defined as the number of malaria confirmed cases (by RDT or microscopy), linked to study clusters by place of residence, per 1000 population per year, using routine data from health facilities serving the study population (e.g., by name of village of residence) and cluster population sizes for the denominator. This outcome is assessed daily at routine health facilities and dispensaries (Mali and Kenya only).
3. Parity as a proxy for daily female vector mosquito survival—this outcome is defined as the non-parous proportion, e.g., the proportion of freshly-caught, non-blood-fed female adult *Anopheles* spp. mosquitoes captured during Human Landing Catches which have never been gravid as determined by the method of Detinova. The outcome is assessed in a sub-sample of clusters, houses, and nights on a monthly basis.
4. Mosquito abundance—The number of adult *Anopheles* spp. captured in CDC UV Light traps.
5. Sporozoite rate—The number of adult female *Anopheles* spp*.* captured via HLC or CDC UV light traps found to be sporozoite positive by anti-circumsporozoite protein (α-CSP) enzyme-linked immune-sorbent assay (ELISA) divided by the number of adult female *Anopheles* spp. tested in ELISA assay.
6. Human landing/biting rate—The number of adult female *Anopheles* spp. captured via HLC divided by the number of person-nights (days) of HLC collection.
7. Entomological inoculation rate—The human landing/biting rate (6) multiplied by the sporozoite rate (5) multiplied by 365 days per year to yield annualized EIR. It should be noted that all EIR estimates will be expressed in terms of annual rates.

## Statistical principles

### Confidence intervals and *p*-values

The trial is generally intended to control type-I error to less than 5%. As such, given the planned interim analyses at each trial site, type-I error will be controlled using an Haybittle-Peto boundaries as discussed above. The main trial results (treatment efficacy estimates) will be presented with 95% confidence intervals and two-sided *p*-values.

### Adherence and protocol deviations

Since the intervention is deployed on a group basis rather than individually, adherence definitions will take account of this. Standard adherence will be defined as the intention to treat a cluster of residences with ATSBs, as randomized. Individual adherence will be defined based on ATSBs present at individuals’ households. Both individual and cluster-level adherence measures will be defined and pre-categorized prior to final analysis and used to categorize the per-protocol trial population.

The per-protocol analysis populations will be defined as those living in intervention clusters where ATSB was deployed and replaced according to the planned schedule. Clusters where more than 1-month delay in ATSB deployment occurred or where substantial deployment of ATSB into control areas occurs (e.g., deployment consistent with distribution of ATSB to control areas) will be removed from the per-protocol analysis population.

Standard protocol deviations will be considered reportable/summarizable when clusters refuse placement of ATSB or have been treated/not treated contrary to their randomization assignment and providing initial study consent. Additionally, protocol deviations will be considered to have occurred if ATSB replacement visits for an entire cluster by the study team are delayed by more than 3 weeks from the expected timeline according to study planning.

Protocol deviations related to failure to deliver or replace ATSB will be summarized in the final trial reports as well as incorporated into the calculation of adherence.

### Analysis populations

There are two analysis populations for the primary outcome assessment: These are the intention-to-treat population and the per-protocol analysis population. The intention-to-treat population consists of all eligible individuals recruited and consented to participate in the study. The primary analysis will be conducted on the intention-to-treat population. Per-protocol analysis populations will be those eligible, recruited, and consented individuals whose adherence at cluster level meets the adherence standard. Additional household-level per-protocol analysis may be conducted consisting of ATSB deployment at the household level consistent with randomization assignment.

### Multiplicity

While the trial tests multiple secondary outcomes, no adjustment will be made for multiplicity because the trials each have two arms and a single primary outcome. Additionally, each trial is powered and run independently and as such no adjustment for multiplicity on account of the three trials is being made. Secondary outcomes are assumed to be on the same causal pathway as the primary outcome and as such are also not adjusted for multiplicity of testing since these are expected to relate to the same hypothesis.

### Trial population

The trial population, as a whole, consists of all de facto and *de jure* residents present in intervention and control clusters (and associated buffer areas where applicable) during the study period. The population to be sampled for outcome assessment considers several additional criteria for inclusion in the cohort studies as outlined below. The clusters for the trials are circumscribed geographic areas usually representing from one to a few villages or in some cases in Zambia, geographically identified parts of villages. Clusters generally represent somewhere from 100 to 400 households in size and widely vary in geographic area. Individuals greater than 18 years of age will provide individual consent. For individuals aged 6 months to less than 18 years of age, consent will be sought from the parent or guardian of the child. For children greater than 6 years and less than 18, oral assent will be sought from the child.

### Screening data

Since the trial is conducted as a cluster randomized study, no individual screening is conducted. Trial areas will be enumerated prior to cohort enrollment and the enumeration will identify households with residents that meet eligibility criteria for cohort participation and for eligibility in cross-sectional household samples (e.g., eligible aged children for outcome assessment). Cluster-level screening is anticipated to be conducted during a baseline period in each study site. A larger number of clusters than planned for the final study power will be included in each site (~ 10% extra clusters). These clusters will be included in baseline data collection but excess clusters will be excluded prior to randomization. Exclusion will consider the following criteria: malaria prevalence and incidence defined as per primary and secondary trial outcomes with a specific aim to exclude any clusters found to have zero or near zero malaria incidence or prevalence in the baseline period or those with dramatically higher incidence/prevalence as compared to other study clusters (e.g., incidence or prevalence > three standard deviations from the mean incidence or prevalence of all baseline clusters will be considered for removal from the study if excess clusters remain after removal of clusters with zero or near zero incidence and prevalence). Additionally, logistical feasibility of implementation will also be considered with clusters in which implementation of intervention or data collection is determined to be impracticable, to be considered for exclusion, or where community-level consent for participation in the trial is refused.

### Eligibility

Eligibility for participation is described in detail in the protocol but in short, the cohort monitoring requires that the individual resides in the study areas within the core sampling areas and additionally is a:

- Household resident
- At least 12 months of age and less than 15 years of age at the time of enrollment (≥ 5 to 15 in Mali, to exclude those covered by Seasonal Malaria Chemoprevention).

And is not a:

- Resident whose home is located within a buffer zone
- Pregnant at the time of cohort enrollment.
- Pregnant at any time during the cohort study.

### Recruitment

Recruitment into the cohort study will be conducted by first completing an enumeration of all households and their members in the study clusters. This enumeration will be used as a sampling frame to select households with eligible individuals for the cohort study. Within each study cluster, a simple random sample of households with eligible individuals will be selected. In Mali and Kenya, a simple random sample of individuals will be selected from census lists. Within clusters, sampling for the cohort study will exclude people living in households within a geographic buffer zone around the perimeter of the cluster. Further details of recruitment are contained in the master trial protocol.

The CONSORT diagram will include at minimum the following elements shown in Table 5.

**Table 5:** CONSORT diagram contents

Cohort study (For each cohort)	Cross-sectional study (each round)
Number of study clusters (by arm)	Number of Study clusters (by arm)
Number of sampled houses (by arm)	Number of Sampled houses (by arm)
Number of consented participants (HHs with participants) (by arm)	Number of consenting houses (by arm)
Number of participants (HHs) randomized to each study arm	Number of completed interviews (by arm)
Number of monthly follow-up visits conducted (by arm)	Number of tested individuals (by arm)
Number of missing HH monthly visits (by arm)	Number of Incomplete HH surveys (by arm)
Number of participants (HH) lost completely to follow-up (by arm)	Number of identified eligible participants not tested (by arm)
Number of participants (HH) completing (by arm)	

### Withdrawal/follow-up

It is anticipated that there will be approximately 20% LTFU withdrawal from each cohort. This is accounted for in the sample size calculations. Level of non-participation in the cross-sectional household surveys is expected to be 10–20%. LTFU will be summarized by arm and by cluster.

### Baseline patient characteristics

The study anticipates summarizing a number of baseline participant characteristics at the individual, household, and cluster levels. Table 6 lists these minimum baseline participant characteristics and the expected summary measures which will be summarized in the cohort and cross-sectional surveys.

**Table 6:** Baseline patient characteristics

Characteristic	Cohort summary measure	Cross-sectional summary measure
**Cluster level**		
** Number of clusters**	*N*	*N*
** Cluster size**	Mean *N* HH (total HH)	Mean *N* HH (total HH)
** Cluster size**	Mean *N* residents (total *N*)	Mean *N* residents (total *N*)
** Cluster size (sampling areas)**	Mean *N* residents (total *N*)	Mean *N* residents (total *N*)
** Cluster size (buffer zones)**	Mean *N* residents (total *N*)	Mean *N* residents (total *N*)
** Baseline incidence**	Mean Incidence rate of clinical malaria in baseline cohort per person month (variance)	Mean incidence rate of clinical malaria in baseline cohort per person month (variance)
** Baseline prevalence**		Proportion positive by RDT for *P. falciparum* at baseline
**Household level**		
** HH size**	Mean *N* residents (SD)	Mean *N* residents (SD)
** LLIN ownership**	Proportion HH with ≥ 1 LLIN (first interview)	Proportion HH with ≥ 1 LLIN
** LLIN ownership**	Proportion HH with ≥ 1 LLIN per 2 residents (first interview)	Proportion HH with ≥ 1 LLIN per 2 residents
**Individual characteristics**		
** Age**	Mean age (SD)	Proportion under five
** Sex**	Proportion female	Proportion female
** HH size**	Mean hh size of participant’s HH (SD)	Mean hh size of included hh (SD)
** Net use**	Proportion slept under the net night before the survey	Proportion (tested population) slept under the net night before the survey

## Analysis

### Outcome definitions

The primary outcome measure is the incidence rate of clinical malaria cases assessed among people aged 12 months to less than 15 years (≥ 5 to 15 in Mali). A clinical case is defined as having an axillary temperature of ≥ 37.5 °C or self-reported fever within the past 48 h, plus a positive malaria RDT. Incidence rate is defined as the total number of incident malaria cases divided by the total person-time observed among each cohort. Outcome assessment will be conducted on each cohort participant monthly. As malaria treatment drugs will be administered to all positive clinical cases (fever + positive RDT) after monthly case ascertainment, each positive (treated) participant will have 2 weeks of the following month of observation time subtracted from their at-risk person-time to account for the prophylactic effect due to sustained antimalarial drug concentration and hence not being at risk of infection. In individuals who are symptomatic and have a positive RDT test in the month following a positive diagnosis of malaria via RDT and treatment, a positive RDT in the following month may indicate persistence of antigen in the blood after effective treatment rather than true reinfection. In such cases, PCR or microscopy results for a *Plasmodium falciparum* infection will be used to resolve if the positive RDT is a result of persistent antigenemia or a true infection (reinfection/recrudescence). In Mali, only microscopy will be used to resolve such cases. In Kenya and Zambia, PCR results will be used where available, and otherwise microscopy. Where the RDT and either the PCR or microscopy results are both positive in month two and the patient meets the other clinical criteria (patent fever or history of fever in the previous 48 h), these observations will be treated as new clinical cases. To keep field procedures unambiguous, a blood slide will be taken whenever a positive RDT is recorded in Mali. Temporary absences from the study area not resulting in failure to ascertain monthly outcomes will not be considered as reducing individual exposure time. Absences greater than the testing interval (1 month ± 5 days) and/or resulting in the failure to ascertain a monthly test result will be removed from the exposure time—meaning that exposure will only be considered to start 1 month prior to the most recent test result.

In summary:

- If a participant is symptomatic and positive by RDT, they are treated and the subsequent 2 weeks of follow-up time are censored.
- If in the next month the participant is also symptomatic and again positive by RDT, they will be treated and PCR or microscopy will be used to determine if they are considered a case of persistent antigenemia or a true new clinical case
- If PCR or microscopy in month two is positive, they are considered to have contributed the person-time between the previous visit and this visit less than 2 weeks and they are considered to contribute a second case to the numerator; two more weeks of follow-up will be censored following the second positive. In Mali, a person who is a malaria case on the day they re-enter the study does not contribute to the number of cases as no follow-up is associated with the case, i.e., they contribute neither to the numerator nor the denominator until they have contributed follow-up.
- If PCR or microscopy is negative, then contributed follow-up time between the previous visit and the second visit with the second positive RDT is included (minus 2 weeks) and only one case is included in the numerator; however, two more weeks of follow-up time are censored after the second RDT positive (due to the required treatment).

### Secondary outcomes

- 1. RDT infection prevalence

Prevalence of patent malaria infection detected by RDT among participants aged 6 months and older is calculated as the number of eligible, consenting participants with positive RDT results divided by the number of eligible, consenting participants with valid RDT results, collected during the cross-sectional survey (rolling survey in Kenya).

- 2. Passive incidence

Incidence rate of passively reported clinical malaria among participants of all ages, defined as the number of malaria confirmed cases (by RDT or microscopy), linked to study clusters by place of residence, per 1000 population per year, using routine data from health facilities with patients linked to study clusters (i.e., by name of the village of residence) and cluster population sizes for the denominator. Cluster population sizes will be calculated based on the number of HH residents identified in the cluster area (core only where possible/relevant) during the census/enumeration. Malaria-confirmed cases will include only those given a diagnosis of blood test (RDT or microscopy) confirmed malaria (ICD-10-M B50-54 and subcodes).

- 3. Parity

The parity outcome is the non-parous proportion: the proportion of freshly caught, non-blood-fed female adult *Anopheles* spp. mosquitoes captured during human landing catch which have never been gravid (are parous) as determined by the method of Detinova (1962). The outcome is assessed in a sub-sample of clusters, houses, and nights. Data will be disaggregated by species (and/or sub-species) with species determination made by taxonomic key and PCR where necessary. Mosquitoes will be classified as parous or non-parous. Mosquitoes with inconclusive results will be excluded from the analysis of parity.

- 4. Mosquito abundance

The number of adult *Anopheles* spp*.* captured in CDC UV light traps per night per trap. The outcome is assessed in a sub-sample of clusters, houses, and nights. Data will be disaggregated by species (and/or sub-species) with species determination made by taxonomic key and PCR where necessary. Mosquitoes with inconclusive speciation will be included in total *Anopheles* spp. abundance calculations but excluded from any species-specific analyses.

- 5. Sporozoite rate

The number of adult female *Anopheles* spp. captured via HLC or CDC UV light trap found to be sporozoite positive by anti-circumsporozoite protein (α-CSP) enzyme-linked immune-sorbent assay (ELISA) divided by the number of adult female *Anopheles* spp*.* tested in ELISA assay. The outcome is assessed in a sub-sample of clusters, houses, and nights. Data will be disaggregated by species (and/or sub-species) with species determination made by taxonomic key and PCR where necessary. Mosquitoes with inconclusive speciation will be included in total *Anopheles* spp. sporozoite rate calculations but excluded from any species-specific analyses. Mosquitoes with inconclusive α-CSP ELISA results will be excluded from all calculations.

- 6. Human landing/biting rate

The number of adult female *Anopheles* spp. captured via HLC divided by the number of person-nights (days) of HLC collection. The outcome is assessed in a sub-sample of clusters, houses and nights. Data will be disaggregated by species (and/or sub-species) with species determination made by taxonomic key and PCR where necessary. Mosquitoes with inconclusive speciation will be included in total *Anopheles* spp. landing rate calculations but excluded from any species-specific analyses. Data will also be disaggregated by indoor versus outdoor collection location.

- 7. EIR

For each month, the month-specific human landing/biting rate will be multiplied (6) by the month-specific sporozoite rate (5) to yield month-specific EIRs. Month-specific EIRs will be summed over the months of the year to yield the number of infectious bites expected in each year. This is a calculated outcome and will be disaggregated by species (and/or sub-species). Results will always be presented in terms of annualized EIR (e.g., number of expected infectious bites per person per year) even when the EIR estimate is made for specific months or other periods.

## Analysis methods

### Primary outcome

The primary unadjusted analysis will be conducted on the intention-to-treat analysis population without adjustment for any anticipated confounding variables as these are considered to be balanced due to randomization. The analysis of the primary outcome, cumulative clinical incidence of malaria, will be analyzed using a multi-level (variance components model) constructed on a generalized linear model framework with a Poisson likelihood and a log link function. Random intercepts will be included for each study cluster and study arm will be included as a fixed effect coded categorically as 0 for arm A and 1 for arm B. The analyst will be blinded to the true assignment until the allocation code is broken. The model will take the form below where *y*_*ij*_ is incidence at the individual level (*i* indexes individuals within clusters and* j* indexes clusters), *α* is the global intercept, *X*_*ij*_ is the arm assignment for individual *i* in cluster *j*, *β*_*arm*_ is the arm effect to be estimated, *u*_*j*_ are random intercepts for the cluster and *exposure*_*i*j_ is the person time at risk for individual *i* in cluster *j*, *λ*_*ij*_ refers to the *E(y*_*ij*_*|u*_*j*_*)*, and *σ* is the standard deviation of the random intercept distribution:

$$\mathrm{log}E\left({y}_{ij}|{u}_{j}\right)=\alpha +{X}_{ij}^{arm}{\beta }_{arm}+{u}_{j}+\mathrm{log}({exposure}_{ij})$$

where the likelihood is of the form:

$${y}_{ij}\sim Pois({\lambda }_{ij})$$

And the random intercepts are assumed to follow a normal distribution:

$${u}_{j}\sim N(0,{\sigma }^{2})$$

Results will be presented as the incidence rate ratio (IRR), corresponding 95% confidence interval, and *p*-value based on the *z*-statistic. The primary outcome will also be checked for the distributional assumption that the mean and variance of the outcome are similar after conditioning on cluster (e.g., are the within-cluster mean and variance similar). If variance is substantially larger, a negative binomial likelihood will be considered.

### Covariate adjusted analysis of the primary and secondary outcomes

Adjusted analyses will be carried out on the primary and secondary outcomes to determine whether the estimate of treatment-effect is affected by the inclusion of additional covariables. The prespecified covariates will be developed and tested prior to final analysis but specific to each site. For the primary and secondary outcomes, one additional analysis will include all covariables which are used in restricted randomization with variables treated exactly as specified in randomization. Because these variables cannot be fully prespecified until the restricted randomization is complete, the full specification of these covariables cannot yet be made. However, these analyses will be prespecified for the primary outcome prior to data lock and the statistical analysis plan for each trial site will be updated to reflect these analyses. Examples of prespecified covariates that may be included in the adjusted analyses are described in Table 7 which will be finalized prior to data lock.

**Table 7:** Proposed covariables

Variable	Categorization (if applicable)	Analysis	Analysis population
Baseline prevalence	Calculated at cluster level	Clinical incidence, prevalence	ITT, per-protocol
Baseline incidence	Calculated at cluster level	Clinical incidence, prevalence	ITT, per-protocol
Rainfall (anomaly)	Summarized monthly at cluster level (lagged one month preceding) as anomaly	Clinical incidence, prevalence	ITT, per-protocol
Season		Clinical incidence, prevalence	ITT, per-protocol
Year	One vs. Two	Clinical incidence, prevalence	ITT, per-protocol
Age	Under 60 months vs. greater than 60 months	Clinical incidence, prevalence	ITT, per-protocol

### Subgroup analysis of the primary outcome

We will perform a series of subgroup analyses according to the list of subgroups in Table 8*.* Imputation for these baseline missing covariates (see the section “Missing data”) will be carried out before categorizing. Assessment of the homogeneity of treatment effect by a subgroup variable will be conducted by the inclusion of the treatment, subgroup variable, and their interaction term as predictors in the adjusted models of primary outcome, and the *p*-value presented for the interaction term. If the *p*-value is less than 0.05, we will present separate effect estimates and confidence intervals for each category of the sub-group variable.

**Table 8:** Planned sub-group analyses

Subgroup name	Categorization	Rationale
Housing type	Closed eaves vs. Non-closed eaves	House structure may act as effect modifier by eliminating indoor biting risk independent of ATSB deployment
Gender	Male vs. Female	Behavioral and occupational difference may act as effect modifier; to demonstrate equity of the intervention effect
One month lagged rainfall (total m per m^2^ previous month)	High vs. low (≥ mean for study site (country) vs. < mean for study site (country))	High levels of absolute rainfall may reduce impact of ATSB by increasing environmental carrying capacity for mosquito population
Season	High vs low (four continuous months of the year with the highest clinical malaria incidence at local health facilities during the trial) vs. eight months with lower incidence	(Kenya only)
Age	≤ 60 months of age vs > 60 months of age	Behavioral differences by age may act as effect modifier
Baseline prevalence	High vs. low (≥ median cluster prevalence vs. < median cluster prevalence)	Local endemicity may act as an effect modifier

## Secondary outcomes

### Prevalence outcomes

The prevalence of malaria infection among participants aged 6 months and older, detected by RDT, will be analyzed using a multi-level (variance components model) constructed on a generalized linear model framework with a Bernoulli likelihood and a logit link function. Random intercepts will be included for each study cluster, and the study arm will be included as a fixed effect coded categorically as 0 for arm A and 1 for arm B. The analyst will be blinded to the true assignment until the results are presented. The model will take the form below where *p*_*ij*_ is the probability of positivity at the individual level (*i* indexes individuals within clusters and* j* indexes clusters), *α* is the global intercept, *X*_ij_ is the arm assignment for individual *i* in cluster *j*, *β*_*arm*_ is the arm effect to be estimated, *u*_*j*_ are random intercepts for the cluster, and *σ* is the standard deviation of the random intercept distribution:

$$\mathrm{logit}({p}_{ij})=\alpha +{X}_{ij}^{arm}{\beta }_{arm}+{u}_{j}$$

where the likelihood is of the form:

$${y}_{ij}\sim Bernoulli({ p}_{ij})$$

And the random intercepts are assumed to follow a normal distribution:

$${u}_{j}\sim N(0,{\sigma }^{2})$$

Model results will be presented as the estimates of *e*^*α*^ and the odds ratio above and the standard deviation or variance of the random effects distribution. 95% confidence intervals for the odds ratio and *e*^*α*^ estimates as well as *z*-statistics and *p*-values for each coefficient will be presented.

### Routine clinical incidence

The incidence of clinical malaria obtained from passive case detection will be analyzed as total incidence using a generalized linear model framework with a Poisson likelihood and a log link function. The incidence will be summed for all months of follow-up within each study cluster, and the study arm will be included as a fixed effect coded categorically as 0 for arm A and 1 for arm B. Exposure will be the population of the cluster as assessed during enumeration. The analyst will be blinded to the true assignment until the results are presented. The model will take the form below where *y*_*i*_ is the total incidence at the cluster level where only aggregated data is available (*i* indexes clusters), *α* is the global intercept, *X*_i_ is the arm assignment for cluster *i*, *β*_*arm*_ is the arm effect to be estimated, *exposure*_*i*_ is the person time at risk for cluster *i*, and *λ*_*ij*_ refers to the *log E(y*_*ij*_*|u*_*j*_*).*:

$$\mathrm{log}E({y}_{i})=\alpha +{X}_{i}^{arm}{\beta }_{arm}+\mathrm{log}({exposure}_{i})$$

where the likelihood is of the form:

$${y}_{i}\sim Pois({\lambda }_{ij})$$

Model results will be presented as the estimates of ${e}^{\alpha }$ and incidence rate ratios above and the standard deviation or variance of the random effects distribution. 95% confidence intervals for the IRR and ${e}^{\alpha }$ estimates as well as *z-*statistics and *p*-values for each coefficient will be presented. Results will be presented as incidence rates and incidence rate ratios along with their associated 95% confidence intervals, and *p*-values.

The outcome will also be checked for the distributional assumption that the mean and variance of the outcome are similar after conditioning on a cluster (e.g., are the within-cluster mean and variance similar); if the variance is substantially larger, a negative binomial likelihood will be considered.

Where individual-level data is available for this outcome, a similar approach will be followed but instead focused on cumulative incidence and using a variance components model. The model will take the form below where *y*_*ij*_ is incidence at the individual (*i* indexes individuals within clusters and* j* indexes clusters), *α* is the global intercept, X_ij_ is the arm assignment for individual *i* in cluster *j*, *β*_*arm*_ is the arm effect to be estimated, *u*_*j*_ are random intercepts for the cluster and *exposure*_*i*j_ is the person time at risk for individual *i* in cluster *j*, *λ*_*ij*_ refers to the *log E(y*_*ij*_*|u*_*j*_*)*, and *σ* is the standard deviation of the random intercept distribution:

$$\mathrm{log}E\left({y}_{ij}|{u}_{j}\right)=\alpha +{X}_{ij}^{arm}{\beta }_{arm}+{u}_{j}+\mathrm{log}({exposure}_{ij})$$

where the likelihood is of the form:

$${y}_{ij}\sim Pois({\lambda }_{ij})$$

And the random intercepts are assumed to follow a normal distribution:

$${u}_{j}\sim N(0,{\sigma }^{2})$$

Results will be presented as the incidence rate ratio (IRR), corresponding 95% confidence interval, and *p*-value based on the *z*-statistic. The primary outcome will also be checked for the distributional assumption that the mean and variance of the outcome are similar after conditioning on a cluster (e.g., are the within-cluster mean and variance similar) and if variance is substantially larger a negative binomial likelihood will be considered.

### Parity

Daily female vector mosquito survival determined by parity is the main entomological outcome of the trial. The primary analysis will be conducted using parity data at the individual mosquito level with a multi-level (variance components model) constructed on a generalized linear model framework with a Bernoulli likelihood and a logit link function. Random intercepts will be included for each entomological study cluster and for each sampling household, and the study arm will be included as a fixed effect coded categorically as 0 for arm A and 1 for arm B. A simple model will first be considered as an unadjusted analysis which only includes fixed effects for study arm as described, and nested random effects for household and study cluster and an intercept. A more fully adjusted model will also be used for analysis to account for the complex sampling design by which mosquitoes are captured for parity analysis. This model will include fixed effects for collection location (indoors vs. outdoors), time since intervention, and calendar month as a seasonality adjustment. Additional random effects will be considered for the catch team/HLC individual. The models will generally take the form below where *p*_*ij*_ is the probability of parity at the individual mosquito level (*i* indexes individual mosquitoes within clusters and* j* indexes clusters), *α* is the global intercept, *X*^*arm*^_*ij*_ is the arm assignment for individual *i* in cluster *j*, *β*_*arm*_ is the arm effect to be estimated, *X*^*indoors*^_*ij*_ represents the individual mosquito being caught indoors, *β*_*indoors*_ is the effect of being indoors on parity relative to collection happening outside, *X*^*time*^_*ij*_ represents a measure of the continuous time since the start of the trial, and *β*_*time*_ is meant to capture an overarching time trend; this variable can also be interacted with the study arm fixed effect to produce an estimate of the difference in time trend by study arm. *X*^*month*^_*ij*_ represents a series of monthly dummy variables in which individual mosquitoes were caught, and *β*_*month*_ represents the series of monthly intercepts, intended to capture seasonal variation in parity. *u*_*j*_ are random intercepts for the cluster, *σ* is the standard deviation of the cluster random intercept distribution, *h*_*k*_ are random intercepts for houses, and *σ*_*h*_ is the standard deviation of the household random intercept distribution:

$$\mathrm{logit}({p}_{ij})=\alpha +{X}_{ij}^{arm}{\beta }_{arm}+{X}_{ij}^{indoors}{\beta }_{indoors}+ {X}_{ij}^{time}{\beta }_{time}+\sum_{month=1}^{11}{X}_{ij}^{month}{\beta }_{month}+{u}_{j }+{h}_{k}$$

where the likelihood is of the form:

$${y}_{ij}\sim Bernoulli({ p}_{ij})$$

And the random intercepts are assumed to follow a normal distribution:

$${u}_{j}\sim N(0,{\sigma }^{2})$$

$${h}_{k} \sim N\left(0,{\sigma }_{h}^{2}\right)$$

Model results will be presented as the estimates of *α* and the odds ratio for the arm above and the standard deviation or variance of the random effects distributions. 95% confidence intervals for the odds ratio and *α* estimates as well as *z*-statistics and *p*-values for each coefficient will be presented.

Analysis based on cluster summaries will also be considered. All parity measurements within each cluster will be summarized as a single proportion. The cluster estimates of the proportion parous will be compared across arms using Student’s *t*-test. Results will be presented as mean parity and standard deviation of parity as well as *t*-statistic and *p*-value. 95% CIs for mean parity will also be presented for each arm.

### Mosquito abundance

The analysis of data on mosquito abundance derived from capture of adult *Anopheles* spp. mosquitoes via CDC UV light traps placed indoors and outdoors near houses overnight will be constructed on a generalized linear model framework with a Poisson likelihood and a log link function. Random intercepts will be included for each entomological study cluster and study arm will be included as a fixed effect coded categorically as 0 for arm A and 1 for arm B. A simple model will first be considered an unadjusted analysis which only includes fixed effects for study arm as described, and cluster-level random effects and an intercept. Autoregressive terms may also be considered with appropriate lags determined by temporal partial auto-correlation functions. The model will take the form below where *y*_ij_ is the count of adult *Anopheles* spp. mosquitoes caught at the individual trap night (*i* indexes individual trap nights within clusters and* j* indexes clusters), *α* is the global intercept, *X*^*arm*^_*ij*_ is the arm assignment for individual *i* in cluster *j*, *β*_*arm*_ is the arm effect to be estimated, *X*^*indoors*^_*ij*_ represents the trap-night observation being indoors, *β*_*indoors*_ is the effect of being indoors on mosquito density/abundance relative to collection happening outside, *X*^*month*^_*ij*_ represents a series of monthly dummy variables in which individual mosquitoes were caught, and *β*_*month*_ represents the series of monthly intercepts. *u*_*j*_ are random intercepts for the cluster and *exposure*_*i*j_ is the number of trap nights corresponding to the particular *y*_*ij*_ observation (generally this will be equal to one (where it does equal one for all observations the log(*exposure*_*ij*_) term may be omitted)) for trap night *i* in cluster *j*, *λ*_*ij*_ refers to the *log E(y*_*ij*_*|u*_*j*_*)*, and *σ* is the standard deviation of the random intercept distribution:

$$\mathrm{log}E\left({y}_{ij}|{u}_{j}\right)=\alpha +{X}_{ij}^{arm}{\beta }_{arm}+{X}_{ij}^{indoors}{\beta }_{indoors}+ \sum_{month=1}^{11}{X}_{ij}^{month}{\beta }_{month}+{u}_{j}+\mathrm{log}({exposure}_{ij})$$

where the likelihood is of the form:

$${y}_{ij}\sim Pois({\lambda }_{ij})$$

And the random intercepts are assumed to follow a normal distribution:

$${u}_{j}\sim N(0,{\sigma }^{2})$$

Results will be presented as the incidence rate ratio (IRR), corresponding 95% confidence interval, and *p*-value based on the *z*-statistic. This outcome will also be checked for the distributional assumption that the mean and variance of the outcome are similar after conditioning on cluster (e.g., are the within-cluster mean and variance similar); if the variance is substantially larger, a negative binomial likelihood will be considered.

### Sporozoite rate

Sporozoite rate or the proportion of adult female *Anopheles* spp. which are sporozoite positive and captured during the trial will be analyzed using a multi-level (variance components model) constructed on a generalized linear model framework with a Bernoulli likelihood and a logit link function. Random intercepts will be included for each study cluster and the study arm will be included as a fixed effect coded categorically as 0 for arm A and 1 for arm B. A simple model will first be considered an unadjusted analysis which only includes fixed effects for the study arm as described and cluster-level random effects and an intercept. A more fully adjusted model will also be used for analysis to account for the complex sampling design by which mosquitoes are captured for α-CSP ELISA. This model will include fixed effects for the capture method (HLC vs. CDC light trap), collection location (indoors vs. outdoors), time since intervention, and calendar month as a seasonality adjustment. Additional random effects will be considered for the catch team/HLC individual. The models will generally take the form below where *p*_*ij*_ is the probability sporozoite positivity at the individual mosquito level (*i* indexes individual mosquitoes within clusters and* j* indexes clusters), *α* is the global intercept, *X*^*arm*^_*ij*_ is the arm assignment for individual *i* in cluster *j*, *β*_*arm*_ is the arm effect to be estimated, *X*^*HLC*^_*ij*_ represents the individual mosquito being caught by HLC, *β*_*HLC*_ is the effect of HLC catch on sporozoite rate relative to CDC light trap, *X*^*indoors*^_*ij*_ represents the individual mosquito being caught indoors, *β*_*indoors*_ is the effect of being indoors on parity relative to collection happening outside, *X*^*time*^_*ij*_ represents a measure of the continuous time since the start of the trial, and *β*_*time*_ is meant to capture an overarching time trend; this variable can also be interacted with the study arm fixed effect to produce an estimate of the difference in time trend by study arm. *X*^*month*^_*ij*_ represents a series of monthly dummy variables in which individual mosquitoes were caught, and *β*_*month*_ represents the series of monthly intercepts, intended to capture seasonal variation in sporozoite rate. *u*_*j*_ are random intercepts for the cluster and *σ* is the standard deviation of the random intercept distribution:

$$\mathrm{logit}({p}_{ij})=\alpha +{X}_{ij}^{arm}{\beta }_{arm}+{X}_{ij}^{HLC}{\beta }_{HLC}+{X}_{ij}^{indoors}{\beta }_{indoors}+ {X}_{ij}^{time}{\beta }_{time}+\sum_{month=1}^{11}{X}_{ij}^{month}{\beta }_{month}+{u}_{j}$$

where the likelihood is of the form:

$${y}_{ij}\sim Bernoulli({ p}_{ij})$$

And the random intercepts are assumed to follow a normal distribution:

$${u}_{j}\sim N(0,{\sigma }^{2})$$

Model results will be presented as the estimates of *α* and the odds ratio above and the standard deviation or variance of the random effects distribution. 95% confidence intervals for the odds ratio and α estimates as well as *z*-statistics and *p*-values for each coefficient will be presented. Sporozoite rate will be directly estimated as the predicted probability of being sporozoite positive in each month when captured via HLC and in each study arm and indoors and outdoors. 95% prediction intervals for sporozoite rate will also be presented.

### Human landing rate

The analysis of data on human landing/biting rate derived from the capture of adult *Anopheles* spp. mosquitoes via HLC conducted indoors and outdoors near houses overnight will be constructed on a generalized linear model framework with a Poisson likelihood and a log link function. Random intercepts will be included for each study cluster, and the study arm will be included as a fixed effect coded categorically as 0 for arm A and 1 for arm B. A simple model will first be considered an unadjusted analysis which only includes fixed effects for the study arm as described, and cluster-level random effects and an intercept. Additional random effects will be considered for catch date, household, and/or HLC “catcher” and autoregressive terms may also be considered with appropriate lags determined by temporal partial auto-correlation functions. The model will take the form below where *y*_*ij*_ is the count of adult *Anopheles* spp. mosquitoes landing on an individual catcher during a specific night (*i* indexes individual catch-nights within clusters and* j* indexes clusters), *α* is the global intercept, *X*^*arm*^_*ij*_ is the arm assignment for individual *i* in cluster *j*, *β*_*arm*_ is the arm effect to be estimated, *X*^*indoors*^_*ij*_ represents the catch-night observation being indoors and *β*_*indoors*_ is the effect of being indoors on human landing relative to collection happening outside, *X*^*month*^_*ij*_ represent a series of monthly dummy variables in which individual mosquitoes were caught and *β*_*month*_ the series of monthly intercepts. *u*_*j*_ are random intercepts for the cluster and *exposure*_*i*j_ is the number of catch-nights corresponding to the particular *y*_*ij*_ observation (generally this will be equal to one (where it does equal one for all observations the log(*exposure*_*ij*_) term may be omitted)) for catch-night *i* in cluster *j*, *λ*_*ij*_ refers to the *log E(y*_*ij*_*|u*_*j*_*)* and *σ* is the standard deviation of the random intercept distribution:

$$\mathrm{log}E\left({y}_{ij}|{u}_{j}\right)=\alpha +{X}_{ij}^{arm}{\beta }_{arm}+{X}_{ij}^{indoors}{\beta }_{indoors}+ \sum_{month=1}^{11}{X}_{ij}^{month}{\beta }_{month}+ {u}_{j}+\mathrm{log}({exposure}_{ij})$$

where the likelihood is of the form:

$${y}_{ij}\sim Pois({\lambda }_{ij})$$

And the random intercepts are assumed to follow a normal distribution:

$${u}_{j}\sim N(0,{\sigma }^{2})$$

Results will be presented as the incidence rate ratio (IRR), corresponding 95% confidence interval, and *p*-value based on the *z*-statistic. This outcome will also be checked for the distributional assumption that the mean and variance of the outcome are similar after conditioning on cluster (e.g., are the within cluster mean and variance similar); if the variance is substantially larger, a negative binomial likelihood will be considered. Human landing rate will be taken to be the predicted mean landing catch per day in each month disaggregated by arm, and indoors vs. outdoors. 95% prediction intervals will also be calculated.

### EIR

The analysis of the entomological inoculation rate will utilize data derived from capture of adult *Anopheles* spp. mosquitoes caught via HLC or CDC light trap indoors or outdoors only and will follow similar principles to the analysis of total sporozoite-positive mosquitoes. The analysis will be based on Student’s *t*-test. For this analysis, estimates of EIR will be made independently for each cluster by calculating an estimated annual EIR within each cluster according to the following formula.

$$EIR= \sum_{i=1}^{n}{30s}_{i}\left\{\begin{array}{c}\frac{\sum_{j=1}^{m}{b}_{ij}}{\sum_{j=1}^{m}{d}_{ij}};if \sum_{j=1}^{m}{d}_{ij}>0\\ 0;otherwise\end{array}\right.$$

where EIR equals the number of infected bites per person night per year and *n* represents the number of months of the year. Where collections are not made during the full calendar year because the malaria transmission season is assumed to be short and infectious bites are not expected outside of the transmission season, zero will be substituted for the estimated number of infectious bites per person-day during these months as shown in the formula above. In the formula above, *b* represents the number of mosquitoes captured via HLC on a catch person-night *j* during month i*, s* represents the estimated sporozoite rate for each cluster in month *i*, *d* represents the number of person catch-days for person catch night *j* in month *i* (which will generally be equal to one), and finally, *m* represents the total number of observations (person catch-nights) of HLC conducted. EIR within each cluster will be summarized as a single annualized EIR estimate post-intervention. The cluster estimates of the EIR will be compared across arms using a Student’s *t*-test. Results will be presented as mean annualized EIR and standard deviation of annualized EIR as well as *t*-statistic and *p*-value. 95% CIs for mean parity will also be presented for each arm. Should the distribution of EIR be substantially non-normal a non-parametric test such as the Mann–Whitney *U*-test may be considered.

## Additional analyses

### Individual pooled analysis across sites

Individual pooled analysis across the three trial sites (countries) will be conducted collectively following the completion of all three trials. This analysis will follow similar statistical principles to each analysis specified above. The pooled analysis will likely include a study site specified as a “fixed” effect to allow for examination of effect modification by site. Factors related to malaria prevalence such as housing density, and density of ATSB coverage in addition to others will be examined as possible determinants of the outcomes or modifiers of ATSB effect. Additionally, a standard individual patient data meta-analysis is expected to be conducted using combined data from all sites. Heterogeneity of results from each site will be examined and this will be used to determine if pooling data and joint estimation of effect size are appropriate or if data should be treated only independently by trial site.

## Missing data

### Missing outcome data

Significant effort will be made to reduce missing outcome data by revisiting cohort households multiple times and pre-scheduling follow-up visits where possible. When missing data does arise due to failed monthly outcome assessment, no imputation will be used. Missing outcomes due to participant absence will result in censoring (removal of the previous period of follow-up time if there is a missing outcome). They will also have 2 weeks of the next period follow-up time removed as per the definition of the primary outcome. Two sensitivity analyses will be carried out for the primary outcome. These will be the last observation carried forward (e.g., an assumption that a clinical malaria case identified at the last time point observed would represent subsequent new clinical cases (and follow-up time removal) at each missing time point or that the absence of a clinical case at last observation would indicate no clinical cases observed at any missing time points and full follow-up time). This analysis is consistent with a true intention to treat protocol. A second sensitivity analysis will be to assume that all missing values would have resulted in negative findings thus imputing zero extra unobserved clinical cases across both study arms and assuming full follow-up time. These analyses will only be applied to the intention-to-treat analysis population because the per-protocol study population already assumes that full follow-up (all outcome assessments) occurred. Full reporting of the fraction of missing outcome assessments by study arm will be conducted for the intention-to-treat study population.

### Missing co-variates

Missing baseline covariates (as defined in the SAP prior to data lock) will be imputed using simple imputation methods in the covariate-adjusted analysis based on the covariate distributions, should the proportion of missing values for a particular covariate be less than 5%. For a continuous variable, missing values will be imputed from random values from a normal distribution with mean and standard deviation calculated from the available sample. For a categorical variable, missing values will be imputed from random values from a uniform distribution with probabilities *P*_1_, *P*_2_, … *P*_k_ from the sample. Seed for the imputation will be pre-set as an 8-digit number based on the date of analysis and documented in all scripts relying on pseudo-random number generators. If the missing values for a covariate are ≥ 5%, then they will be imputed using Markov chain Monte Carlo (MCMC) methods [6].

### Harms

The main risks associated with the intervention are the risk of ingestion of the bait + toxicant by humans, animals, and/or non-target arthropods—particularly the local pollinator insect (bee) population. To mitigate ingestion risk for humans and other mammals bittering agents (Bitrex™) have been added to ATSBs to reduce likelihood of ingestion of ATSBs. Pre-trial studies suggest that interaction between pollinators and ATSBs is insignificant and therefore ATSBs are not a risk to NTOs. As the main harms are not expected to be encountered by study participants there is no formal plan for statistical analyses of harms to study participants. Continued monitoring of trial sites for misuse or product loss will be conducted and these data will be reviewed by the DSMB but they will not be formally analyzed statistically. Unexpected harm may occur during the course of trial and will be considered in reviews and by DSMB though no formal analysis is planned.

### Statistical software and other trial-specific management procedures

Statistical software and hardware platforms may vary by trial site. Reporting of statistical analysis will include specific details of software platform, including language, version, and details of any additional libraries used in analysis. Each trial will also develop a trial-specific SAP and maintain trial-specific standard operating procedures, trial master files, and statistical master files.

### Supplementary Information

**Additional file 1.**

**Additional file 2.**

## Data Availability

The datasets analyzed during the current study will be made available from the corresponding author and/or the study site principal investigator upon reasonable request.
